# Pulmonary Involvement in Proteus Syndrome: Clinical and Imaging Correlates in a Rare Case

**DOI:** 10.1155/crra/9944074

**Published:** 2026-01-08

**Authors:** Cody Reid Johnson, Syed Muhammad Awais Bukhari, Amit Gupta

**Affiliations:** ^1^ Department of Radiology, University Hospitals Cleveland Medical Center, Cleveland, Ohio, USA, uhhospitals.org; ^2^ Department of Biomedical Engineering, Case Western Reserve University, Cleveland, Ohio, USA, case.edu

**Keywords:** bullous changes, hamartoma, Proteus, pulmonary, pulmonary nodules, vascular abnormalities

## Abstract

Proteus syndrome is an uncommon, sporadic disorder characterized by progressive and heterogeneous overgrowth of tissues, resulting in distorted and asymmetric development. In most individuals, Proteus syndrome has minimal to no manifestations at birth but progresses during childhood and adolescence. Clinical manifestations of the disease include isolated asymmetric hemihyperplasia, isolated asymmetric macrodactyly, subcutaneous masses, plantar and palmar cerebriform fibrous overgrowth, exostoses, epidermal nevi, and scoliosis. Cardiothoracic structures are less commonly involved, and the manifestations include cystic lung changes, pulmonary thromboembolism and varicosities, and pulmonary nodules. Patients with Proteus syndrome have an increased risk of early death due to deep venous thrombosis and pulmonary embolism. We report a case of an adult female who was diagnosed with Proteus syndrome at the age of 5 years who had multiple pulmonary manifestations of the disease.

## 1. Introduction

Proteus syndrome was first formally described in 1979 and was subsequently termed “Proteus syndrome” by Wiedemann et al. [1]. The disorder is characterized by the asymmetric overgrowth of multiple tissues with mosaic phenotypic expression [2]. The etymology of the syndrome derives from the Greek god Proteus, known for the ability to change form, which is analogous to the variable manifestations of Proteus syndrome [3].

Proteus syndrome is a rare disorder with features of asymmetric hypertrophy and overgrowth of multiple body tissues with an incidence of less than 1 out of 1 million live births [4, 5]. The onset of overgrowth classically begins in infancy, is progressive throughout life, and involves multiple body tissues with varying degrees of severity in affected individuals. Due to the numerous different phenotypes of the disorder, diagnosis can be challenging [6]. Reportedly common and diagnostic features include cerebriform connective tissue nevi, epidermal nevi, asymmetric/disproportionate overgrowth, certain neoplasms, vascular malformations, typical facial phenotypes (hyperostosis, hemimegalencephaly, and craniosynostosis), dysregulated adipose tissue, and lung cysts [7].

Cardiothoracic findings in Proteus syndrome are not uncommon. Patients with Proteus syndrome have an increased risk of early death resulting from pulmonary thromboembolism, pneumonia, and postoperative complications [7]. The increased incidence of thromboembolism may be due to more numerous corrective surgeries that these patients undergo or due to vascular malformations that predispose to thrombus formation [8]. Pulmonary manifestations of Proteus syndrome include expanded, emphysematous airspaces and the formation of multiple lobulated bullae; pulmonary nodules; dilatation of the pulmonary veins/varicosities; and bandlike areas of scarring. Both calcified and noncalcified nodules can be present. In this case report, we present a patient with multiple pulmonary manifestations and a reported diagnosis of Proteus syndrome. We intend to emphasize the distinctive features of pulmonary abnormalities on chest computed tomography (CT) images.

### 1.1. Case Report

A woman in her 30s with a known case of Proteus syndrome with AKT1 mutation came to our institution for a routine follow‐up. She had experienced progressive skeletal overgrowth on the right side during the course of her life, including in the right hand, multiple right fingers, right elbow, right patella, right second and fourth toes, and right leg and foot (Figure 1). She has had multiple surgeries for her condition in the past. Her surgical history includes partial right middle lobe lung resection by video‐assisted thoracic surgery (VATS) and right thoracotomy for hemothorax, craniotomy, multiple finger amputations, laparoscopic assisted total vaginal hysterectomy for multiple endocrine polyps and cervical dysplasia, bilateral oophorectomy at age 6.25 years for pathology‐diagnosed serous cystadenomas with focal, nuclear atypia (epithelial membrane antigen positive, carcinoembryonic antigen negative) of both ovaries, appendectomy, and epiphysiodesis in her right leg. She also has asymmetric hyperostosis of the right aspect of the frontal bone. The patient has no known brain parenchymal abnormalities.

Figure 1Pictures of the (a) upper and (b) lower limbs taken after the patient′s consent. Right‐sided partial gigantism is clearly demonstrated. Please note that the patient′s right index and ring fingers were surgically amputated at age 5 for progressive overgrowth, with the resultant biopsy confirming cartilaginous hamartomas in them.

**Figure:**
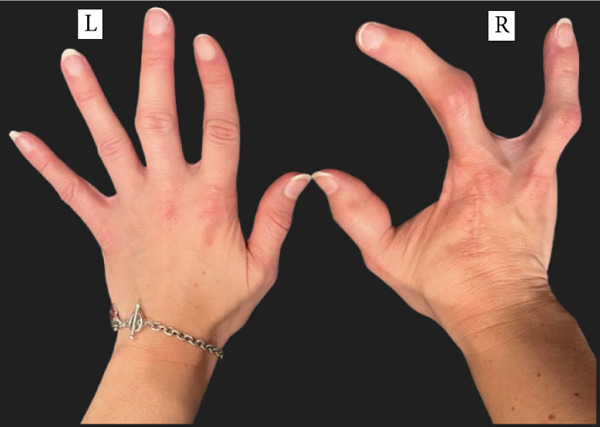
(a)

**Figure:**
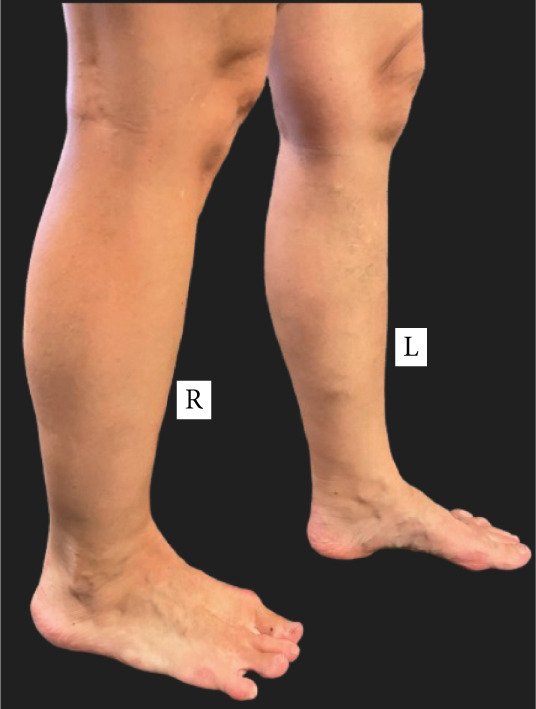
(b)

On routine evaluation, a pulmonary nodule was noted on the right upper lobe (Figure 2a,b). The patient had a remote history of recurrent bronchitis and pulmonary infection. She had no reported pulmonary symptoms at the time of the visit. Vital signs were stable at the time of examination.

Figure 2Pulmonary parenchymal abnormalities in a woman in her 30s with Proteus syndrome. (a) Coronal CT image of the chest in lung window setting demonstrates a rounded nodule in the right upper lobe (red arrow) and bullous changes in the right lower lobe (tan arrow). (b) Axial CT image through the upper lung shows the nodule more clearly (red arrow) with internal fat attenuation (Mean HU: 47) seen on the corresponding soft tissue window (1b insert). (c) Axial image through the lower chest shows the complete extent of the bullous lung parenchymal changes (tan arrows).

**Figure:**
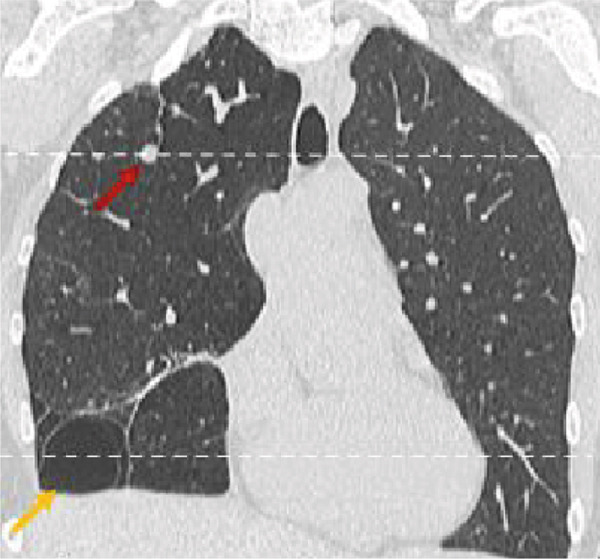
(a)

**Figure:**
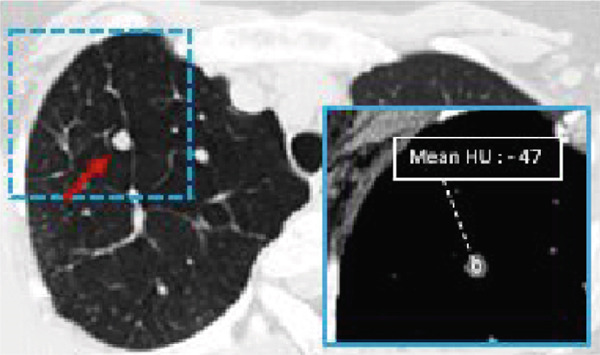
(b)

**Figure:**
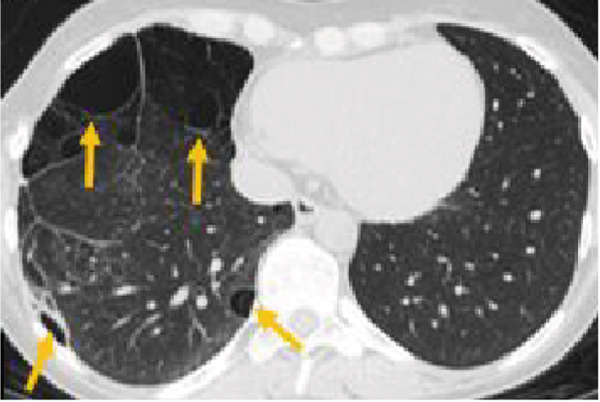
(c)

A 7‐mm pulmonary nodule was noted in the right upper lobe on noncontrast multidetector computed tomography (NC‐MDCT) of the chest (Figure 2a,b). Areas of attenuation within the intrapulmonary nodule demonstrated Hounsfield units (HU) of −47.75, consistent with internal fat and suggestive of pulmonary hamartoma (Figure 2b). The lungs also demonstrated peripheral bullous changes with areas of irregular air space enlargement, greatest in the right lower lobe (Figures 2a,c). Coronal maximum‐intensity projection (MIP) images depicted an anomalous course of the right superior lobe vein (Figure 3). The right superior lobe vein most commonly drains directly into the left atrium after joining obliquely with the right middle lobe vein; however, in this case, it has a more vertical course before joining the right middle lobe vein.

Figure 3Pulmonary vascular abnormalities in a woman in her 30s with Proteus syndrome. A coronal maximum‐intensity projection (MIP) (a) CT image of the chest in lung window setting demonstrates an atypical (vertical) course of the right superior lobe vein (red arrows). (b) Axial MIP CT image through the apical lungs demonstrates multiple venous varicosities in the apical right upper lobe (tan arrows).

**Figure:**
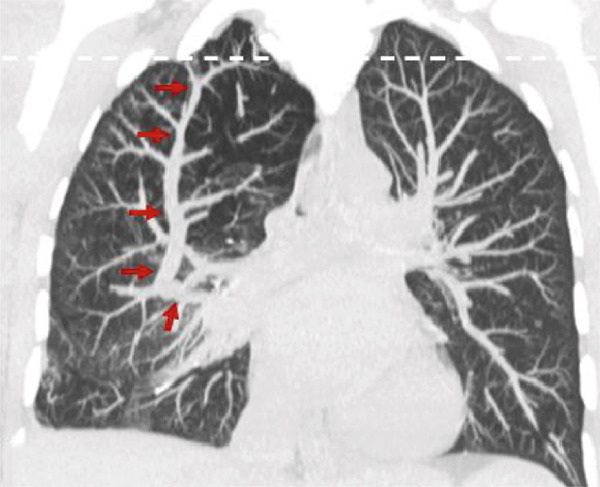
(a)

**Figure:**
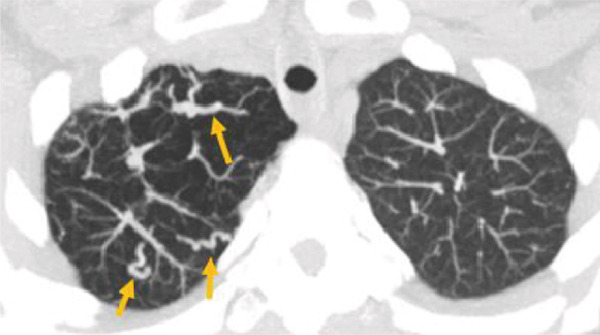
(b)

The patient subsequently had no reported pulmonary complaints. She did eventually undergo facetectomy and discectomy of her thoracic spine secondary to neurogenic pain and recovered well from that procedure. She presented to the ER approximately 6 months later with a chief complaint of light‐headedness and underwent a CT pulmonary angiogram, which was negative for pulmonary embolism and demonstrated stability of the bullous changes and the known nodule. She continues to do well from a pulmonary standpoint.

## 2. Discussion

The diagnostic criteria for Proteus syndrome include evaluation for a pathogenic variant of the AKT1 gene, which contributes to the mosaicism found in the disease as well as evaluating for certain common phenotypic features. A complete list of the diagnostic criteria is beyond the scope of this case report, but the classic features of the disease include asymmetric, disproportionate overgrowth; organ overgrowth; bullae or cysts of the lungs; various tumors; dysregulated adipose tissue; facial phenotypes; and vascular malformations [9]. Each of these features is given a point score and a score of ≥ 10 points with an AKT1 mutation or a score of ≥ 15 points without an AKT1 mutation can yield the diagnosis [9].

According to one retrospective study, common cardiothoracic features of Proteus syndrome include hyperlucent lung parenchyma (50%), pulmonary nodules (50%), bandlike scarring (56%), and pulmonary venous dilation (62%) [9]. Pulmonary disease may be unnoticed in some patients because other features of Proteus syndrome are phenotypically more prominent [10]. Although they are not frequently reported in the literature, some nodules may represent pulmonary hamartomas given the overall disease manifestations and presumed pathophysiology. Although the nodule in our case was not biopsied and there is no pathology available, the internal fat attenuation is most consistent with a pulmonary hamartoma.

The distribution of bullae in Proteus syndrome can be unilateral or bilateral. There does not appear to be a predilection for any specific lung lobe. The etiology of the bullous lung malformations in Proteus syndrome has not been fully elucidated. It can be inferred that abnormal overgrowth of various pulmonary tissues might be related to the development of pulmonary emphysematous changes.

On chest radiographs, the cystic airspace disease in patients with Proteus syndrome may be appreciated though more subtle changes require CT for optimal evaluation and may be useful for screening younger patients before onset of pulmonary symptoms. Pulmonary vascular anomalies were also present in our case and, in general, they may be more common than previously thought as they are often asymptomatic [11].

Furthermore, vascular abnormalities may mimic consolidation due to pneumonia or atelectasis on chest radiographs and may hence be misdiagnosed. In the rare event that pulmonary vascular anomalies become symptomatic, they can thrombose or, potentially, rupture [11]. Thus, pulmonary venous abnormalities should be suspected when a chronic opacity is detected on serial radiographs, particularly when associated with other clinical manifestations of Proteus or other overgrowth syndromes.

## Ethics Statement

The authors declare that appropriate written informed consent was obtained for the publication of this manuscript and accompanying images.

## Consent

Written informed consent was obtained from the patient for the publication of this case report and accompanying images.

## Conflicts of Interest

The authors declare no conflicts of interest.

## Author Contributions

Cody Reid Johnson participated in writing the paper. Syed Muhammad Awais Bukhari and Amit Gupta participated in reviewing, editing, and finalizing the manuscript.

## Funding

No funding was received for this manuscript.

## Data Availability

The data that support the findings of this study are available from the corresponding author upon reasonable request.
